# Furanoate-Based Nanocomposites: A Case Study Using Poly(Butylene 2,5-Furanoate) and Poly(Butylene 2,5-Furanoate)-*co*-(Butylene Diglycolate) and Bacterial Cellulose

**DOI:** 10.3390/polym10080810

**Published:** 2018-07-24

**Authors:** Marina Matos, Andreia F. Sousa, Nuno H. C. S. Silva, Carmen S. R. Freire, Márcia Andrade, Adélio Mendes, Armando J. D. Silvestre

**Affiliations:** 1 CICECO—Aveiro Institute of Materials, Departmento de Química, Universidade de Aveiro, 3810-193 Aveiro, Portugal; marina.matos@ua.pt (M.M.); nhsilva@ua.pt (N.H.C.S.S.); cfreire@ua.pt (C.S.R.F.); armsil@ua.pt (A.J.D.S.); 2 Laboratory for Process Engineering, Environment, Biotechnology and Energy (LEPABE), Faculdade de Engenharia da Universidade do Porto, Rua Dr. Roberto Frias, 4200-465 Porto, Portugal; mrsa@fe.up.pt (M.A.); mendes@fe.up.pt (A.M.)

**Keywords:** 2,5-furandicarboxylic acid, poly(1,4-butylene 2,5-furandicarboxylate), biobased materials, bacterial cellulose, nanocomposites, mechanical properties

## Abstract

Polyesters made from 2,5-furandicarboxylic acid (FDCA) have been in the spotlight due to their renewable origins, together with the promising thermal, mechanical, and/or barrier properties. Following the same trend, (nano)composite materials based on FDCA could also generate similar interest, especially because novel materials with enhanced or refined properties could be obtained. This paper presents a case study on the use of furanoate-based polyesters and bacterial cellulose to prepare nanocomposites, namely acetylated bacterial cellulose/poly(butylene 2,5-furandicarboxylate) and acetylated bacterial cellulose/poly(butylene 2,5-furandicarboxylate)-*co*-(butylene diglycolate)s. The balance between flexibility, prompted by the furanoate-diglycolate polymeric matrix; and the high strength prompted by the bacterial cellulose fibres, enabled the preparation of a wide range of new nanocomposite materials. The new nanocomposites had a glass transition between −25–46 °C and a melting temperature of 61–174 °C; and they were thermally stable up to 239–324 °C. Furthermore, these materials were highly reinforced materials with an enhanced Young’s modulus (up to 1239 MPa) compared to their neat copolyester counterparts. This was associated with both the reinforcing action of the cellulose fibres and the degree of crystallinity of the nanocomposites. In terms of elongation at break, the nanocomposites prepared from copolyesters with higher amounts of diglycolate moieties displayed higher elongations due to the soft nature of these segments.

## 1. Introduction

The last decades have seen a burgeoning search for more sustainable chemicals, polymers, and materials due to severe environmental concerns and to the announced depletion of fossil resources [1]. In this context, renewable-based chemicals, such as those derived from C5 and C6 biomass sugars, namely the 2,5-furandicarboxylic acid (FDCA), and the polyesters thereof, have been in the spotlight [2]. Some of the most successful examples, due to their promising properties, comparable to fossil-based terephthalate homologues, including poly(ethylene 2,5-furandicarboxylate) (PEF) [3,4], and poly(1,4-butylene 2,5-furandicarboxylate) (PBF) [5,6,7,8,9,10,11,12,13,14], also known as poly(ethylene 2,5-furanoate) and poly(1,4-butylene 2,5-furanoate), respectively. They are expected to replace poly(ethylene terephthalate) (PET) and poly(1,4-butylene terephthalate) (PBT), respectively, on various conventional applications of thermoplastics, such as, for example in packaging materials in the case of PEF or in electronic applications in the case of PBF [2].

Furthermore, FDCA-derived copolyesters have also been extensively studied, with the aim of expanding or refining even further the properties and/or potential applications of their parent homopolymers [7,15,16,17,18,19,20,21,22]. Amongst the wide library of these copolymers, furanoate-aliphatic copolyesters were the most studied [2,15,16,17,21,22,23,24,25,26,27,28,29,30], and those incorporating ether linkages, such as the work of Lotti et al. based on diglycolic acid [25] or of Sousa et al. using poly(ethylene glycol) [16], are particularly interesting. For example, the 100% renewable poly(butylene 2,5-furanoate)-*co*-(butylene diglycolate)s (with 60 to 90 mol % of furanoate moieties), henceforth designated by PBF-*co*-PBDG, are biodegradable and could have an elongation at break of up to four times higher than PBF [25]. In fact, the incorporation of high quantities of soft butylene diglycolate units brings significant improvement in the elongation, but at the expense of the Young’s Modulus (roughly 10 times lower compared to PBF). In terms of gas barrier properties, PBF-*co*-PBDG can exhibit adequate behaviour for packaging materials applications. The oxygen gas transmission rate (GTR) varied between 111–193 cm^3^ m^−2^ d^−1^ bar^−1^ [25].

More recently, (nano)composite and hybrid materials based on furanoate-based polymeric matrices have also been developed [31,32,33,34,35,36], although still they are modestly and mostly restricted to PEF. However, the significant properties improvement of the ensuing materials, relevant to their processing and/or application (e.g., crystallisation rate improvement), will predictably foster their rapid development in the near future. PEF-derived hybrid materials were prepared by compounding PEF with inorganic fillers, added during the synthesis of the polymer. For example, Bikiaris and co-workers [32] demonstrated that the in situ preparation of PEF/SiO_2_ and PEF/TiO_2_ hybrid materials, during solid state polymerization, lead to slightly higher molecular weight PEF due to the presence of the SiO_2_ or TiO_2_ nanoparticles. Lotti et al. [35] also synthesised hybrid materials based on PEF containing either graphene oxide or multi walled carbon nanotubes (non-functionalised, or functionalised with –COOH or –NH_2_ groups). Differential scanning calorimetry analysis indicated that all the fillers acted as nucleating agents for the PEF crystallisation, albeit in a different extent. Other works are focused on PEF-derived (nano)composites with cellulose fibres [31,36], organically modified montmorillonite clays and sepiolite clays [33,34]. Of particular interest is the work carried out by the Guigo and Sbirrazzuoli group on PEF composites using small quantities of nanocrystalline cellulose (around 4 wt %) and prepared via twin screw extrusion [31] or solvent casting [36]. These composites have enhanced crystallisation properties in the presence of the fibres, namely faster crystallisation [31] and nucleating effects [36], despite some compatibility problems associated with the hydrophilic nature of pristine cellulose compared to PEF homopolyester [36]. Adding to this, nanocellulose fibres, in particular bacterial cellulose (BC) produced by *Gluconoacetobacter sacchari* bacterial strain at high purity, due to its nanofibrillar structure having unique physical and chemical properties as a nanocomposite [37,38], including optically transparency and high mechanical strength [39]. However, to the best of our knowledge, BC has never before been used in the preparation of furanoate-based nanocomposites. In this vein, this study presents a new family of fully bio-based nanocomposites, prepared from a series of PBF-*co*-PBDG copolyesters, or PBF, and modified bacterial cellulose previously treated with heterogeneous acetylation (to improve compatibility with the thermoplastic matrices). These PBF-*co*-PBDG and PBF-acetylated-BC nanocomposites were chosen as a case study for the broader development of furanoate-based nanocomposites, and in particular for their potential to enhance their mechanical properties. The newly prepared nanomaterials were fully characterised through several structural, thermal, and mechanical techniques, as well as in terms of gas permeability, aiming to access their potential use for packaging applications.

## 2. Experimental

### 2.1. Materials

Bacterial cellulose in the form of wet membranes was produced using the *Gluconoacetobacter sacchari* bacterial strain and conventional culture medium conditions, as described elsewhere [40]. 2,5-Furandicarboxylic acid (FDCA, >98%) and 1,1,1,3,3,3-hexafluoro-2-propanol (HFP, >99%) were purchased from TCI Europe NV. Diglycolic acid (DGA, 98%), 1,4-butanediol (BD, 99%), titanium (IV) tert-butoxide (Ti(OBu)_4_, pro-analysis), trifluoroacetic acid, (TFA, 99%) and deuterated trifluoroacetic acid (TFA-d, 99 atom % D) were supplied by Sigma-Aldrich Chemicals Corporation (Sintra, Portugal). Sulfuric acid (H_2_SO_4_, 96%) was supplied by Acros Organic (Geel, Belgium). All chemicals were used as received.

### 2.2. Heterogeneous Acetylation of Bacterial Cellulose

Prior to heterogeneous acetylation, the BC wet membrane was disintegrated using a blender and an Ultra-Turrax equipment (15 min at 20,500 rpm), and solvent exchanged with ethanol and acetone (in triplicate). Heterogeneous acetylation of BC fibres was then carried out following a well-established protocol described elsewhere [39]. Briefly, acetic anhydride (225 mL) was placed in a 500 mL round flask into an ice bath for 20 min, then 1 mL of H_2_SO_4_ was added, and finally the wet BC fibres (≈40 g) were added to the mixture. The reaction was allowed to proceed under stirring for 4 h at 30 °C. The ensuing BC-acetylated fibres (Ac-BC) were filtered and sequentially washed with water, acetone, ethanol, water, and again with ethanol. Finally, Ac-BC nanofibres were Soxhlet-extracted with ethanol for 12 h to remove any residual trace of acetic anhydride or other impurities, and solvent exchanged with acetone followed by chloroform.

### 2.3. Preparation of the Acetylated BC/Poly(Butylene Furandicarboxylate-co-Butylene Diglycolate) Nanocomposites (Ac-BC/PBF-co-PBDG)

#### 2.3.1. Synthesis of PBF-*co*-PBDG Copolyesters and Corresponding Homopolyesters

The polyesters were synthesized via a procedure described elsewhere [18,41]: Fisher esterification of FDCA, esterification, and finally polycondensation reaction. In brief, dimethyl 2,5-dimethylfurandicarboxylate (DMFDC) was first prepared by reacting FDCA (192.2 mmol) with an excess of methanol (364 mL), under acidic conditions (HCl, 15 mL), at 80 °C for 15 h. The reaction mixture was allowed to cool down, and the ensuing white precipitate was isolated by filtration in 70% yield. Secondly, DMFDC and DGA (mol % DMFDC/mol % DGA ≈ 90/10, 75/25, 50/50, 25/75 and 10/90) were mixed with an excess of BD (1.5 mol per 1 mol of DMFDC and DGA) under a nitrogen atmosphere. The temperature was then raised to 110 °C, Ti(OBu)_4_ catalyst (1.4 mmol) added and the temperature was again progressively raised to 200 °C. Here, a slightly different procedure was followed, depending on the polyester being synthesized.

In the case of PBF, PBF-*co*-PBDG-90/10, 75/25, and 50/50 (i.e., those polyesters prepared from higher amounts of DMFDC) the reaction mixture was kept at 200 °C for 4 h. Then, the reaction proceeded by applying a vacuum (ca. 10^−3^ bar) for 1 h. Subsequently, the temperature was raised again to approximately 210 °C and kept at that temperature for more than 4 h. In the other cases of PBDG, PBF-*co*-PBDG-25/75 and 10/90, the period at 200 °C was only 2 h, followed by an additional 2 h period, at 210 °C. In the third-step, the reaction proceeded at 210 °C under vacuum, and then the temperature was raised to 220 °C for 4 h. 

Then, the mixture was purified by dissolving the polymers in TFA (20 mL), pouring in an excess of cold methanol (ca. 1 L), separation by filtration, and drying. The isolation yields of the polymers were ca. 60%, which was in accordance with previous results [16].

#### 2.3.2. Preparation of Ac-BC/PBF-*co*-PBDG Nanocomposites, and Corresponding Homopolyesters Nanocomposites

The nanocomposites were prepared by a well-known solvent casting approach. The polyesters (0.21 g) were mixed with a BC or Ac-BC chloroform dispersion (0.0045 g/mL, 20 mL) under magnetic stirring for 3 h. The mixture was then deposited into a square Teflon mould (6.5 cm^2^) and the films were cast, at room temperature, for a minimum of 15 h, and finally heated at 30 °C, under vacuum, for 12 h to remove any remaining solvent. The ensuing films had a thickness of approximately 0.098 ± 0.001 mm.

### 2.4. Characterisation Techniques

Attenuated total reflectance Fourier transform infrared (ATR FTIR) spectra were obtained using a PARAGON 1000 Perkin-Elmer FTIR spectrometer equipped with a single-horizontal Golden Gate ATR cell. The spectra were recorded after 128 scans, at a resolution of 4 cm^−1^, within a range of 500 to 4000 cm^−1^. ^1^H and ^13^C nuclear magnetic resonance (NMR) spectra were recorded using a Bruker AMX 300 spectrometer, operating at 300 or 75 MHz, respectively. All chemical shifts (δ) were expressed as parts per million, downfield from tetramethylsilane (used as the internal standard). Elemental analyses (C and H) were conducted in triplicate using a LECO TruSpec analyser. The degree of substitution (DS) was estimated through the approach of Vaca-Garcia et al. [42]: DS= (5.13766 − 11.5592 × C)/(0.996863 × C − 0.856277 × *n* + *n* × C) where n and C stand for the number of carbon atoms in the acyl group and for the carbon contents, respectively.

Scanning electron microscopy (SEM) images of the surface and cross-sections of films were acquired using a field emission gun-SEM Hitachi SU70 microscope operating at 4 kV. Samples were deposited onto a sample holder and coated with carbon twice.

X-ray diffraction (XRD) analyses were performed using a Philips X’pert MPD diffractometer operating with CuKα radiation (λ = 1.5405980 Å) at 40 kV and 50 mA. Samples were scanned in the 2θ range of 5° to 50°, with a step size of 0.04°, and a time per step of 50 s.

Differential scanning calorimetry (DSC) thermograms were obtained with a DSC Q100 V9.9 Build 303 (Universal V4.5A) calorimeter from Texas Instruments, using steel DSC pans. Scans were carried out under nitrogen with a heating rate of 10 °C min^−1^ in the temperature range of −90 to 250 °C. Two heating/cooling cycles were repeated. Glass transition (T_g_) was determined using the midpoint approach (second heating trace); melting (T_m_) and crystallisation (T_cc_) temperatures were determined as the maximum of the exothermic crystallisation peak, and the minimum of the melting endothermic peak during the second heating cycle, respectively.

Thermogravimetric analyses (TGA) were carried out with a Setaram SETSYS analyser equipped with an alumina plate. Thermograms were recorded under a nitrogen flow of 20 mL min^−1^ and heated at a constant rate of 10 °C min^−1^ from room temperature up to 800 °C. Thermal decomposition temperatures were taken at the onset of significant weight loss (5%) and at maximum decomposition temperatures from the heated samples (T_d,5%_ and T_d_, respectively).

Tensile tests were obtained with an Instron 5564 tensile testing machine at a cross-head speed of 10 mm min^−1^ using a 500 N static load cell. The tensile test specimens were rectangular strips (50 mm × 10 mm) pre-conditioned for 72 h at 50% humidity and 30 °C. Each measurement was repeated at least five times.

Contact angle (CA_water_) measurements with water were carried out using a Contact Angle System OCA20 goniometer (DataPhysics, Filderstadt, Germany) with SCA20 software using the sessile drop approach, and recorded during 40 s. Water was used as probe liquid, and for each specimen, drops of 3 μL were deposited using a syringe (50 μL) onto the nanocomposite film surface. The error analysis was obtained by the standard deviation of at least five independent determinations.

Permeation measurements were performed in a system that included a membrane cell connected to a tank with a calibrated volume (at the permeate side) and to a gas cylinder (at the feed side). Prior to permeation tests, the films were glued to steel O-rings with an epoxy glue (Araldite^®^ Standard, Huntsman Advanced Materials, Basel, Switzerland); the glue was also applied along the interface of the steel O-ring and the film, as described elsewhere [43]. A sintered metal disc covered with a filter paper was used as support for the film in the test cell. Single gases were tested at 30 °C, where the feed pressure was 1 bar and the permeate pressure was ca. 0.03 bar. The tests were performed in a standard pressure-rise setup using an acquisition program based on LabView^®^ platform (National Instruments, Austin, TX, USA). The permeability towards a pure component *i* was determined accordingly to: $L_{i}=\frac{F_{i}}{\Delta P_{i}/l}$, where $F_{i}$ is the flux of species *i*, $\Delta P_{i}\;$ is the partial pressure difference of species between the two sides of the membrane, and l is the film thickness. The permeability to the pure component was computed from the experimental data as follows: $L_{i}=\frac{lV_{p}v_{M}}{RTA\left(P_{f}-P_{p}\right)}\;\frac{\Delta P_{p}}{\Delta t}$, where $V_{p}$ is the volume of the permeate tank, $v_{M}$ is the molar volume of the gas at normal conditions, *R* is the gas constant, *T* is the absolute temperature, *t* is the time, *A* is the effective permeating area of the film, and $P_{f}$ and $P_{p}$ are the feed pressure and permeate pressure, respectively, and $\Delta P_{p}$ is the permeate pressure increment for the elapsed time Δt.

## 3. Results

### 3.1. From Furanoate-Glycolate Copolyesters to Acetylated Bacterial Cellulose-Based Nanocomposites

**Scheme 1:**
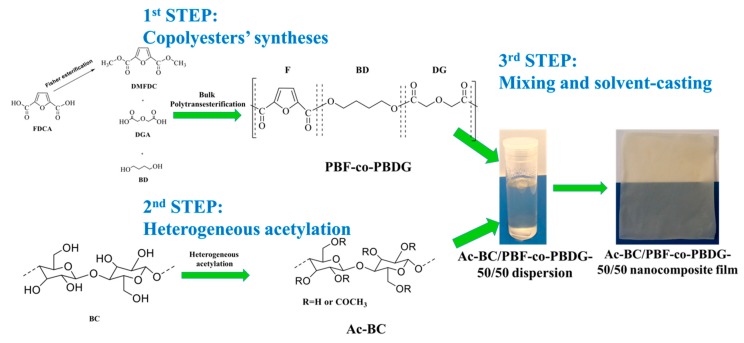
The Ac-BC/PBF-*co*-PBDG composite preparation approach.

A series of Ac-BC/PBF-*co*-PBDGs, Ac-BC/PBF, and Ac-BC/PBDG nanocomposites were developed for the first time following a three-step procedure (Scheme 1).

In the first step, the (co)polyesters were prepared by a conventional bulk polyesterification approach [18,41]. These (co)polyesters spanned from the neat PBF to neat PBDG, and encompassed their copolyesters with different relative furanoate/digycolate amounts (90/10, 75/25, and the never reported 50/50, 25/75, and 10/90 mol %). The selection of these (co)polyesters was based on their promising properties, notably biodegradability and high elongation at break [25], and aiming to further improve their mechanical properties.

In the second step, heterogeneous acetylation of the BC fibres were performed using acetic anhydride. The degree of acetylation (DS) of the Ac-BC was determined using elemental analysis, by the Vaca-Garcia et al. [42] approach, and the resulting value was 0.87.

In the third step, nanocomposite films of each (co)polyester and Ac-BC were obtained by solvent-casting aiming to obtain novel nanomaterials with enhanced mechanical properties. Importantly, this approach could be generally applied to other furanoate thermoplastics as a strategy to improve their mechanical performance, namely to recycled PEF. Recycled thermoplastics lose some of their high-performance mechanical properties, mostly due to a reduction of the molecular weight. However, compounding these thermoplastics with the Ac-BC nanofibres could play a reinforcing role.

The relative amount of (co)polyester/Ac-BC used in this work in the nanocomposites preparation was approximately equal to 70/30 wt %, based on the fact that a minimum of 30 wt % of Ac-BC was required to form the films. For comparison reasons, films of each individual component of the nanocomposites were additionally prepared. Pure Ac-Bc generates a white thin film, but the neat copolyesters did not form films by solvent-casting; in fact, this was consistent with the fact that a minimum of 30 wt % of Ac-BC was needed in order to obtain the nanocomposites films.

For comparison reasons, nanocomposites of non-acetylated BC/PBF-*co*-PBDGs were also prepared following a similar approach. However, these materials were shown to be very heterogeneous; hence they were no further investigated. On the contrary the nanocomposite materials prepared using the Ac-BC fibres were homogeneous and translucent, indicating a good dispersion of the modified BC in the thermoplastic polymeric matrices.

### 3.2. Structure and Morphology

The starting (co)polymers components were studied ^1^H and ^13^C NMR analysis. The main results are recorded in the Supplementary data (Figure S1, and also Tables S1 and S2), and were consistent with previously published data [25]. One important aspect studied, due to the influence on the final properties of the (co)polyesters and consequently also on the related nanocomposites, were the assessment of the real furanoate/diglycolate incorporation (Table S2). Results indicated a trend towards incorporating slightly more diglycolate moieties in the copolymer back-bone than in the initial feed ratio, except for PBF-*co*-PBDG-50/50 copolyester (7 mol % higher than expected).

**Figure 1:**
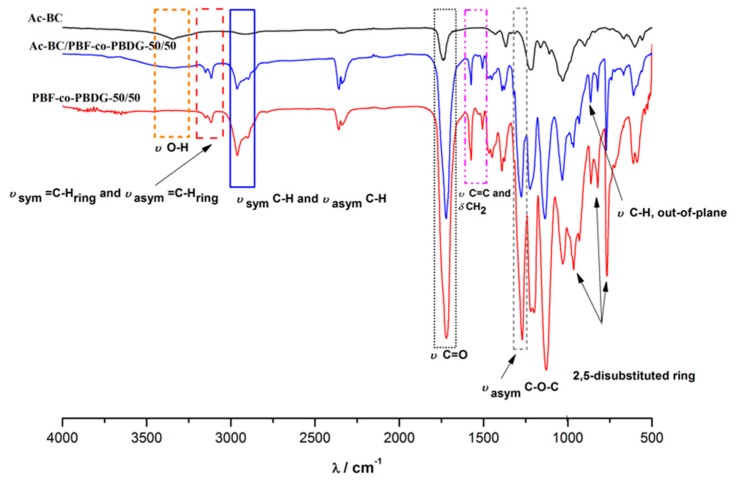
Attenuated total reflectance Fourier transform infrared (ATR FTIR) spectra of Ac-BC/PBF-*co*-PBDG-50/50 nanocomposite and corresponding Ac-BC and PFB-*co*-PBDG-50/50 components.

All furanoate-based nanocomposites and corresponding components (Ac-BC, PBF-*co*-PBDG, PBF, and PBDG polyesters) were also thoroughly characterised by means of ATR FTIR spectroscopy (Figure 1 and Figures S2–S4 of Supplementary data).

The Ac-BC/PBF-*co*-PBDG nanocomposites displayed the typical vibration modes of furanoate-based polyesters [44] and in particular of PBF-*co*-PBDGs: two week bands centred at 3150 and 3115 cm^−1^ arising from the symmetrical and asymmetrical C–H stretching of the furanic ring (ν_sym_ = C–H_ring_ and ν_asym_ = C–H_ring_), two other weak bands near 2962 and 2890 cm^−1^ arising from the symmetrical and asymmetrical C–H stretching characteristics of the methylene groups of the BD and diglycolate moieties (ν_sym_ C–H and ν_asym_ C–H), and a very intense band centred at 1720 cm^−1^, arising from the carbonyl stretching vibration, typical of ester groups (ν C=O). In addition, these spectra also showed a band near 1506 cm^−1^, assigned to both the C=C stretching and CH_2_ in plane deformation (ν C=C, δ CH_2_, respectively), a band near 1263 cm^−1^ arising from the ν_asym_ C–O–C stretching, and several vibrations in the finger print region related to the 2,5-disubstitued ring. The vibrational modes of acetylated BC and those of the polyesters were partially overlapped, as can be confirmed by inspection of the corresponding spectra of Figure 1. However, a distinct feature of the nanocomposites spectra due to the cellulose incorporation was the broad band detected near the 3351 cm^−1^ characteristics of the ν O–H. All of the characteristic vibrational features of Ac-BC and BC precursors are summarised in the Supplementary data (Figure S2).

**Figure 2:**
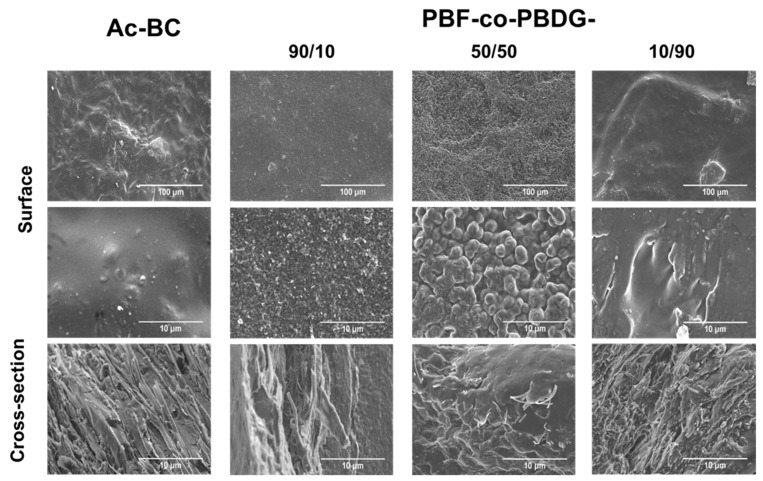
Surface (**top**) and cross-section (**bottom**) SEM micrographs of Ac-BC film and of selected nanocomposite films.

With regards to the morphology, SEM micrographs of the nanocomposites with higher content of diglycolate units (≥50 mol %), collected at different magnifications (Figure 2 and Figure S5 of Supplementary data), showed a smoother and uniform surface, thus indicating enhanced compatibility between the fibres and those polymeric matrices. From this perspective, the amount of Ac-BC used (around 30 wt %) and the heterogeneous acetylation of the cellulose fibres carried out in order to increase the cellulose hydrophobicity (DS ≈ 0.87) and, thus, the compatibility between the modified fibres and the polyesters, was shown to be an adequate approach for obtaining homogeneous nanocomposites, especially in the case of Ac-Bc/PBF-*co*-PBDG-10/90 and -25/75, and Ac-BC/PBDG. A more extensive acetylation of the fibres could, in principle, increase the compatibility of the more hydrophobic furanoate polyesters (such as, PBF, PBF-*co*-PBDG-90/10) [25], but this would also have disrupted the characteristic BC nanostructure and thus would have extensively affected the properties of the ensuing materials. Another, possibility, worth considering in future work, will be the addition of an extra compatibility agent, or even a plasticiser acting also as a compatibility agent. Nevertheless, this would have brought an extra complexity to the data interpretation of the nanocomposite-systems, deviating from the present study as a more in-depth analysis of the basic principles governing cellulose/PBF-*co*-PBDG properties.

It is evident from the cross-section pictures (Figure 2 and Figure S5 of Supplementary data), the presence of Ac-BC nanofibres embedded within the polymeric matrix. Further, these results confirmed that the interfacial adhesion between the Ac-BC fibres and the polymeric matrices was particularly good for the high diglycolate content polyesters, namely PBF-*co*-PBDG-10/90, -25/75, and PBDG.

**Figure 3:**
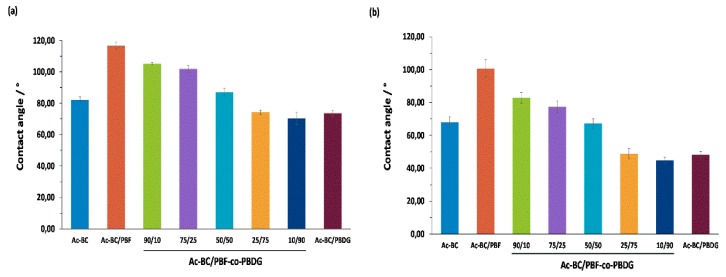
Water contact angles at (**a**) 0 and (**b**) 15 s.

The nanocomposite hydrophobicity was evaluated through water contact angle (CA_water_) measurements at several points in time for 40 s after the water droplet deposition. The main results are displayed in Figure 3 and summarised in Table S3 of Supplementary data.

The CA_water_ decreased drastically over the initial 5 s, and then roughly maintained constant. This behaviour was due to the initial re-orientation of the functional groups at surface of the films, allowing the water drops to spread more easily [45]. Among different nanocomposites, the CA_water_, after 15 s, increased with the increasing furanoate content in the copolyester (from 45 to 100°), mostly due to the hydrophobic character of the furanoate-based polyesters [41]. The Ac-BC film showed an intermediate CA_water_ of approximately 67.9° after 15 s, in accordance with nanofibre affinity to the polyesters, and consequently good dispersion in the thermoplastic matrices, especially PBF-*co*-PBDG-50/50 to -10/90, and PBDG. The wide range of water contact angles covered by these nanomaterials, from highly hydrophobic (ca. 100.57°) to moderate hydrophilic (ca. 44.81°) was an interesting feature worth exploiting in different applications, such as, for example in packaging [25] or textiles.

### 3.3. Cristallinity and Thermal Behaviour

**Figure 4:**
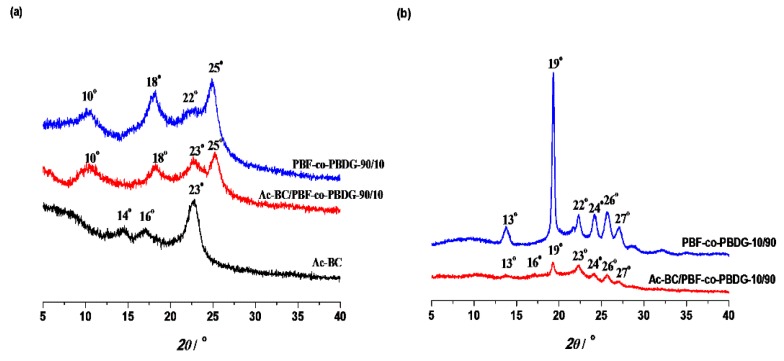
X-Ray diffractograms of: (**a**) Ac-BC/PBF-*co*-PBDG-90/10 nanocomposite film and corresponding Ac-BC film and PBF-*co*-PBDG-90/10 components, and (**b**) Ac-BC/PBF-*co*-PBDG-10/90 nanocomposite film and corresponding Ac-BC film and PBF-*co*-PBDG-10/90 components.

The nature of the crystalline domains of the nanocomposites prepared with a wide range of furanoate/diglycolate copolyesters and with Ac-BC fibres was evaluated by XRD (Figure 4 and Figure S6 of Supplementary Materials).

The nanocomposites prepared with the copolyesters containing a higher amount of furanoate moieties (i.e., PBF-*co*-PBDG-90/10 to 50/50) roughly displayed the typical diffraction pattern of PBF, with strong reflections at 2θ ≈ 18 and 25°, and smaller peaks at 2θ ≈ 10 and 22° [8]. In the case of the diffractogram of the nanocomposite prepared with the copolyester containing the lowest amount of furanoate moieties (Ac-BC/PBF-*co*-PBDG-10/90), the main peaks observed were those typical of PBDG precursors, viz: 2θ ≈ 14, 19, 22, 24, 26, and 27° [25]. These results allowed one to associate the crystalline domain of Ac-BC/PBF-*co*-PBDG-90/10, 75/25, and 50/50 nanocomposites to PBF, whereas in the case of Ac-BC/PBF-*co*-PBDG-10/90, it was essentially related to PBDG. In addition, the XRD diffraction patterns of the nanocomposite films were all naturally related with the copolyesters counterparts (in the form of powder), despite some differences in the sharpness of the reflection peaks, as easily attested by comparing both (Figure S6 of Supplementary data). These results could be associated with the incorporation of Ac-BC fibres into the polymeric matrix and/or due to solvent casting film formation conditions.

In the particular case of Ac-BC/PBF-*co*-PBDG-25/75, a more pronounced effect was noted; indeed, this nanocomposite was amorphous, displaying accordingly on its diffractogram a pronounced amorphous halo cantered at 22°, despite its precursor displaying some crystallinity (see Figure S6 of Supplementary Materials).

Importantly, the thermal and mechanical behaviour of all nanocomposites were influenced by their degree of crystallinity and also by the nature of this domain, as discussed below.

**Figure 5:**
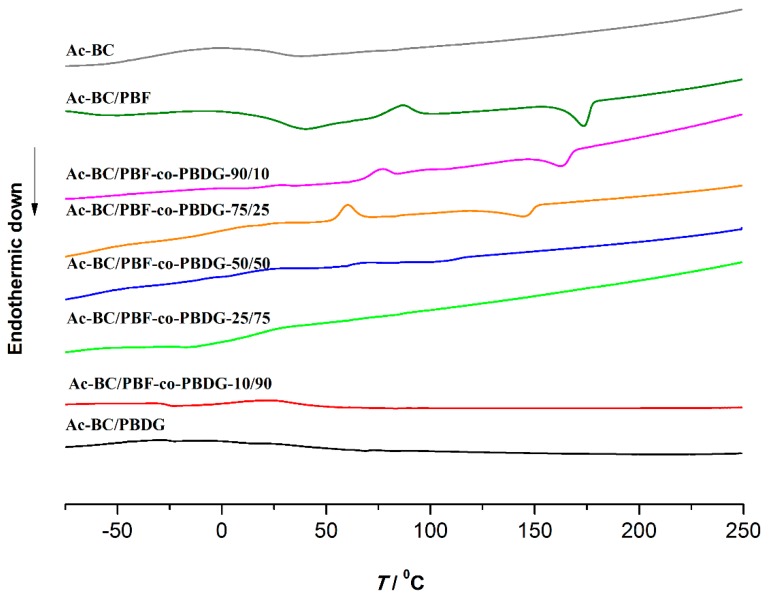
DSC traces of the nanocomposites and Ac-BC.

All Ac-BC/PBF-*co*-PBDG nanocomposites and the corresponding individual components precursors were further characterised in terms of their thermal behaviour through DSC and TGA analyses (Table 1, Figure 5 and, Table S4 and Figures S7–S9 of Supplementary Materials).

The DSC traces of the nanocomposites (Table 1 and Figure 5) were in accordance with the semi-crystalline nature of the nanocomposites or instead with the amorphous character of one of these materials, as XRD results indicated. Therefore, the DSC traces of Ac-BC/PBF, Ac-BC/PBF-*co*-PBDG-90/10, -50/50 and -10/90, and Ac-Bc/PBDG displayed a glass transition (T_g_), followed by a melting (T_m_) event at 46.1 to −24.9 °C, and 173.5 to 61.4 °C, respectively. An additional cold crystallisation (T_cc_) event was also observed after the T_g_ in the cases of Ac-BC/PBF, PBF-*co*-PBDG-90/10 and -75/25, which might be associated to an additional nucleation effect of Ac-BC fibres [46]. The corresponding traces of neat PBF and PBF-*co*-PBDG-90/10 (co)polyesters (Figure S7 and Table S4 of Supplementary Material) did not showed a cold crystallisation event.

In regard to Ac-BC/PBF-*co*-PBDG-25/75, the corresponding thermogram displayed only a step in the baseline at ca. −12.6 °C, attributed to the glass transition temperature, due to its essentially amorphous nature, in agreement with the XRD results.

For all nanocomposites, T_g_ decreased (around 13 °C) with an increased amount of diglycolate units in the copolyester. In the same vein, the T_m_ of the nanocomposites also decreased from 173.5 to 61.4 °C with an increasing amount of soft diglycolate segments. This trend was also observed in the case of neat (co)polyesters prepared in this work (Table S4 of Supplementary Material) and reported elsewhere [25].

In addition, the T_g_ of the nanocomposite films tended to be higher than those obtained for the corresponding (co)polyester component, in agreement with the higher stiffness of the nanocomposites. For example, Ac-Bc/PBF-*co*-PBDG-75/25 had a T_g_ of 25.6 °C, whereas the same parameter for PBF-*co*-PBDG-75/25 was 13.8 °C. In regard to the T_m_, the nanocomposites (Table 1) and the corresponding copolyesters synthesised in this work (Table S4 of Supplementary Material) had very similar results, but they were higher than literature values [25].

The typical TGA thermograms of the newly prepared furanoate nanocomposites (Table 1 and Figure S9 of Supplementary Material) displayed two major characteristic steps at maximum decomposition temperatures (T_d,max_) of 348–362 °C and 376–385 °C. The first step was due to the Ac-BC decomposition and was quite close to that observed for the neat Ac-BC fibres (363.0 °C) and comparable to previously reported results [39]. The other decomposition step was associated with the polyesters enriched fraction and was observed at higher temperatures than the related polyester precursor. For example, in the case of Ac-BC/PBF-*co*-PBDG-50/50 the second T_d,max_ was equal to 380.7 °C, whereas the same parameter was equal to 365.1 °C for the PBF-*co*-PBDG-50/50 copolyester. These results were comparable to those reported for other nanocomposites of Ac-BC and poly(lactic acid) [39].

The nanocomposites were thermally stable up to 238–306 °C (Table 1), indicating a decrease of the stability compared to the related polyesters (360–380 °C) (Table S4 of Supplementary Materials). The same effect was previously reported with PEF/cellulose materials [31]. Additionally, one can notice, on both nanocomposites and the corresponding polyester component, an increase of the T_d,5%_ with the amount of furanoate incorporated into the polymeric matrix backbone. These thermal features enabled the establishment a maximum working temperature of up to 306 °C for the novel nanocomposites.

### 3.4. Mechanical Properties and Permeability Assays for Oxygen

**Figure 6:**
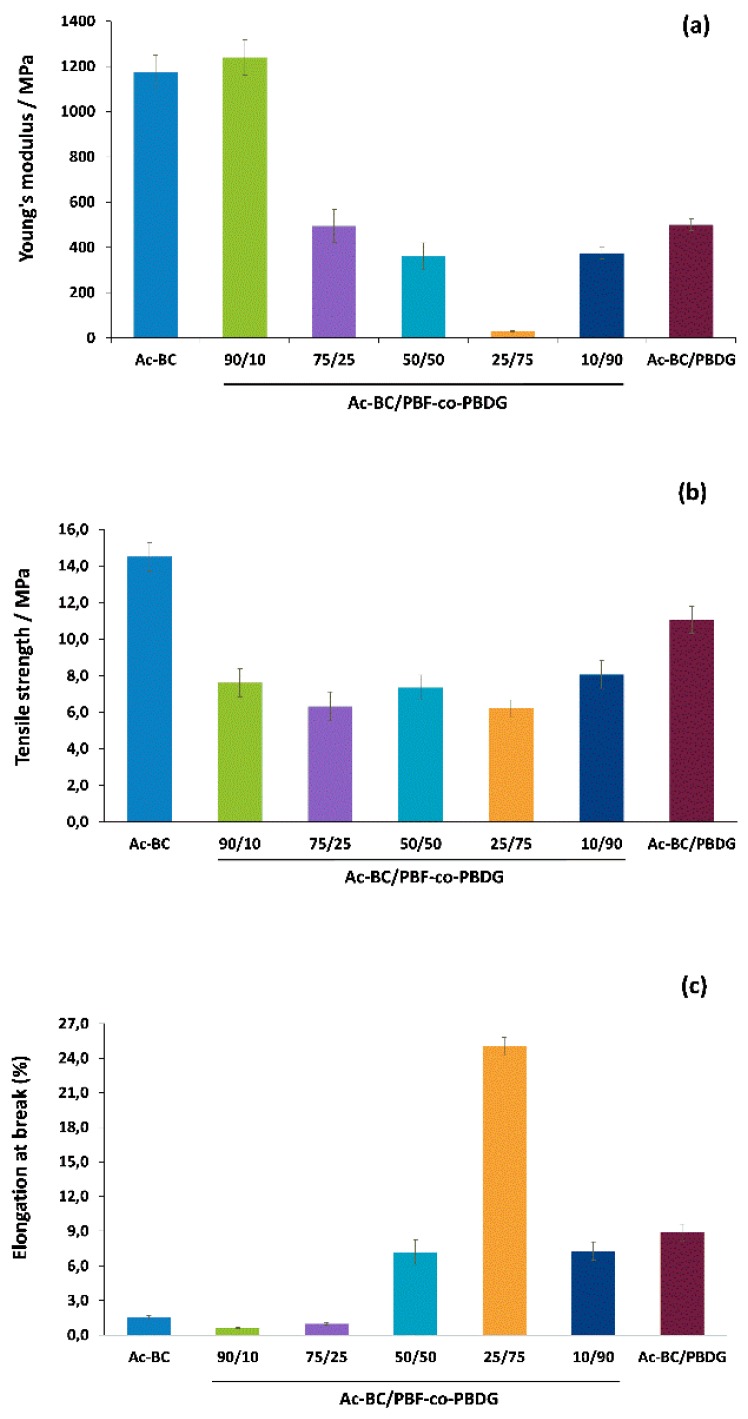
(**a**) Young’s modulus, (**b**) tensile strength and (**c**) elongation at break of the nanocomposites and of the Ac-BC component.

Tensile tests were performed to assess the mechanical performance of these novel furanoate-based nanocomposites and in particular to evaluate the effect of compounding cellulose nanofibres (Ac-BC) with PBF-*co*-PBDG copolyesters. The stress-strain behaviour of Ac-BC/PBF-*co*-PBDGs was revealed to be dependent on a complex interplay of factors, namely the chemical composition of the related (co)polyesters and the presence of nanofibres, as well as the crystallinity of the new materials. The main results are displayed in Figure 6 and summarised in Table S5.

Ac-BC/PBF-*co*-PBDG-90/10 exhibited the highest Young’s modulus, approximately 1239.3 MPa, in accordance with this nanocomposite having the highest amount of rigid furanoate moieties. This result was significantly higher than that previous reported to the related film of the neat copolyester (ca. 373 MPa) [25]. The cellulose fibres played here had a reinforcing role in the nanocomposites, as well as due to the higher crystallinity of the copolyester prepared in this work, thus explaining the Young’s modulus increase.

Importantly, the Young’s modulus of Ac-BC/PBF-*co*-PBDG-90/10 (ca. 1239.3 MPa) was very close to that routinely reported for PBF [11,25,28], and also very near to neat Ac-BC film (ca. 1172.8 MPa). This was a very interesting result because this nanocomposite had good mechanical properties, comparable to those of PBF; but had the advantage of being biodegradable oppositely to PBF [25].

The other nanocomposites showed lower Young’s modulus (ca. 499.9–30.3 MPa), decreasing with decreasing amounts of furanoate units from 75/25 to 25/75, but slightly increasing again to Ac-BC/PBF-*co*-PBDG-10/90 and PBDG. This inverted bell-shape trend behaviour for the first decreasing trend was in agreement with the decrease of stiff furanoate moieties; and for the second increasing trend it was most probably associated with an increase in crystallinity as prompted by the substantial amount of diglycolate segments, and thus a lower degree of randomness (as well as disclosed by XRD analysis). In addition, the nanocomposites Young’s modulus results were typically much higher than those reported for the neat copolyesters films produced by Soccio et al. [25] due to the expectable reinforcing role of the cellulose fibres [39], and due to a higher crystallinity of the herein prepared nanocomposites.

In terms of elongation at break, the nanocomposites prepared from copolyesters with higher amounts of diglycolate moieties displayed higher elongations due to the soft nature of these segments. The highest result was obtained with Ac-BC/PBF-*co*-PBDG-25/75 (ca. 25.02%). Despite this composite having a huge gain in elasticity, especially compared to cellulose fibres, the elongation at break was still lower than those of neat copolyesters [25].

The nanocomposites barrier properties were evaluated in terms of permeability to oxygen, and preliminary results indicated that the nanocomposites and the corresponding (co)polyesters films prepared elsewhere [25] had similar permeabilities towards oxygen. Ac-BC/PBF-*co*-PBDG-90/10 showed to have a permeability to O_2_ that was equal to 3.49 × 10^2^ Barrer, whereas Ac-BC had 1.75 × 10^5^, in accordance with the well-documented [44,47] superior barrier properties of furanoate-based polymers. These were attractive properties worth exploiting for applications within packaging. 

## 4. Conclusions

Furanoate-based (nano)composites using bacterial nano-cellulose was here reported for the first time, revealing great potential to broaden the properties of this material. In the present case study involving Ac-BC/PBF and Ac-BC/PBF-*co*-PBDGs nanocomposites, they were shown to have high stiffness (evaluated by the Young’s modulus, from 30.3 to 1239 MPa) compared to the neat (co)polyesters counterparts. Concomitantly, these nanocomposites still displayed reasonable elasticity (elongation at break) compared e.g., to cellulose or PBF. These properties were only possible by judiciously tailoring the composition of the nanocomposites, especially the critical diglycolate/furanoate amount in the copolyester, as well as by compounding the (co)polyesters with acetylated cellulose (tailoring crystallinity, homogeneity, among other properties). Moreover, the permeabilities to oxygen results were quite attractive, being in the order of magnitude of PBF, justifying further exploitation of these nanocomposites for applications within packaging. 

## Figures and Tables

**Table 1:** Important thermal values obtained from differential scanning calorimetry (DSC) and thermogravimetric analysis (TGA) analyses.

Sample	T_cc_^1^/°C	T_g_^1^/°C	T_m_^1^/°C ^1^	T_d, 5%_^2^/°C	T_d,max_^2^/°C
**Ac-BC/PBF**	86.5	46.1	173.5	323.8	354.7; 384.2
**Ac-BC/PBDG**	-	−24.9	66.1 ^3^	284.0	362.1; 384.0
**Ac-BC/PBF-*co*-PBDG-**					
**90/10**	76.3	25.8	162.9	305.8	354.9; 383.0
**75/25**	60.3	15.2	144.8	300.2	353.9; 376.6
**50/50**	-	−1.8	94.6	297.9	348.2; 380.7
**25/75**	-	−12.6	-	238.8	362.3; 378.6
**10/90**	-	−20.4	61.4 ^3^	293.6	359.8; 384.8

^1^ Determined by DSC from the second heating scan at 10 °C min^−1^. ^2^ Determined by TGA at 20 °C min^−1^. ^3^ Determined by DSC from the first heating scan at 10 °C min^−1^.

## Supplementary Materials

The following are available online at http://www.mdpi.com/2073-4360/10/8/810/s1, Scheme S1: Chemical structures of the triad units of the PBF-*co*-PBDG copolyesters, Figure S1: ^1^H NMR spectra in TFA-d of PBF-*co*-PBDG copolyesters and related PBF and PBDG homopolyesters, Table S1: Main ^1^H NMR resonances of PBF-*co*-PBDG copolyesters and related PBF and PBDG homopolyesters, Table S2: Comparison between the initial molar feed percentage and the real molar percentage of furanoate and diglycolate moieties, Figure S2: ATR FTIR spectra of the acetylated bacterial cellulose (Ac-BC) and of the unmodified bacterial cellulose (BC) fibres, Figure S3: ATR FTIR spectra of PBF-*co*-PBDG copolyesters and of PBF- and PBDG-related homopolyesters, Figure S4: ATR FTIR spectra of all Ac-BC/PBF-*co*-PBDG nanocomposites, Figure S5: SEM micrographs of Ac-BC film and of the nanocomposites of the (a) surface (500 X and 5.0 kX) and (b) cross-section (500 X and 5.0 kX), Table S3: Water contact angles of the composite films measured at several points in time for 40 s, Figure S6: X-ray diffractograms of the (a) neat (co)polyesters and (b) corresponding nanocomposites, Table S4: Important thermal values of the (co)polyesters and Ac-BC obtained by DSC and TGA analyses, Figure S7: DSC traces of the PBF-*co*-PBDGs and related PBF and PBDG homopolyesters.
