# Supplementary material for: Furanoate-Based Nanocomposites: A Case Study Using Poly(Butylene 2,5-Furanoate) and Poly(Butylene 2,5-Furanoate)-co-(Butylene Diglycolate) and Bacterial Cellulose
Source: Polymers (Basel). 2018 Jul 24;10(8):810. doi: 10.3390/polym10080810 (PMC6403708; doi:10.3390/polym10080810)
Supplement: Supplementary file 1 [file polymers-10-00810-s001.pdf]

polymers

Supplementary data for:

Furanoate based nanocomposites: a case study using poly(butylene 2,5-furanoate) and poly(butylene 2,5-furanoate)-co-(butylene diglycolate) and bacterial cellulose

Marina Matos [1], Andreia F Sousa [1,*], Nuno HCS Silva [1], Carmen S R Freire [1], Márcia Andrade [2], Adélio Mendes [2] and Armando J D Silvestre [1]

[1] CICECO – Aveiro Institute of Materials, Departamento de Química, Universidade de Aveiro, 3810-193 Aveiro, Portugal; andreiafs@ua.pt
[2] Laboratory for Process Engineering, Environment, Biotechnology and Energy (LEPABE), Faculdade de Engenharia da Universidade do Porto, Rua Dr. Roberto Frias 4200-465 Porto, Portugal
* Correspondence: andreiafs@ua.pt; Tel.: +351 234 370 200

Table of Contents

1. Structure and morphology 2
1.1 ^{1}H NMR 2
1.2 ATR-FTIR 4
1.3 SEM 5
***2.* Contact angles with water 6**
3. Crystallinity and thermal behaviour 6
3.1 X-ray diffraction (XRD) analysis 6
3.2 Differential scanning calorimetry (DSC) 7
3.3 Thermogravimetric analysis (TGA) 9
3. Tensile tests 11

1. Structure and morphology

1.1 ¹H NMR

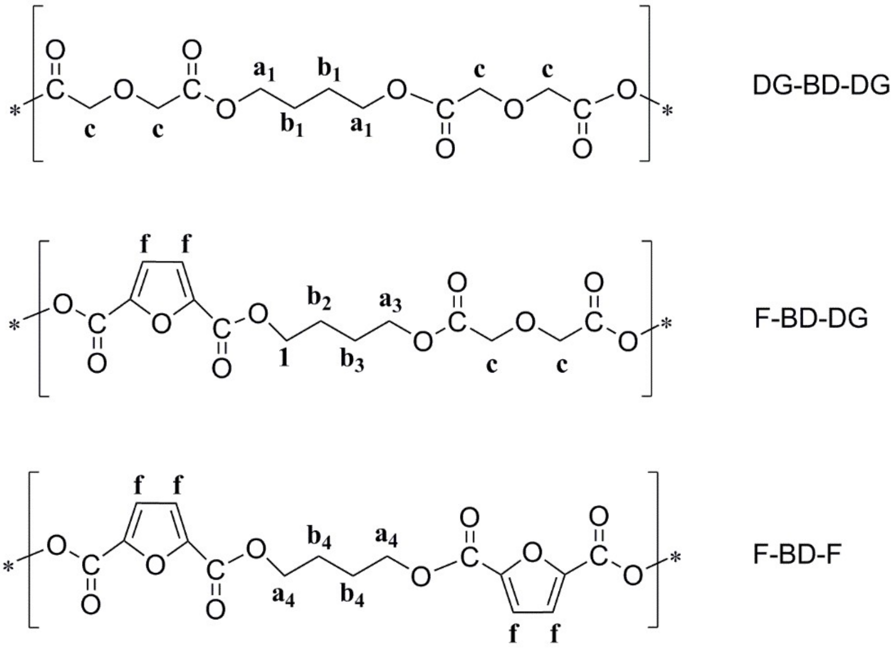

Scheme S1. Chemical structures of the triad units of the PBF-co-PBDG copolyesters.

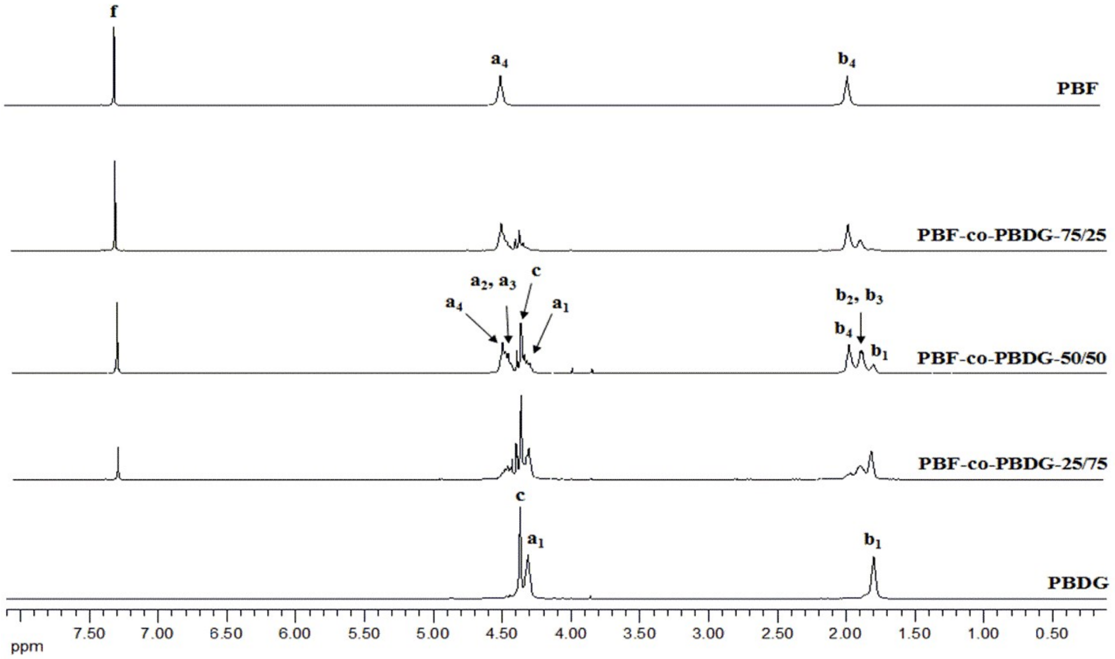

Figure S1. ^{1}H NMR spectra in TFA-*d* of PBF-co-PBDG copolyesters and related PBF and PBDG homopolyesters.

Table S1. Main ^{1}H NMR resonances of PBF-co-PBDG copolyesters and related PBF and PBDG homopolyesters.

δ / ppm	assignment	triads	integration area						
			PBF	PBF-co-PBDG-					PBDG
				90/10	75/25	50/50	25/75	10/90	
**7.30**	**f; C*H* (FDCA)**	**F-BD-F; F-BD-DG**	1.00	1.00	1.00	1.00	1.00	1.00	–
**4.50**	**a_4; OCH_2 (BD)**	**F-BD-F**	2.00	1.85	1.72	1.52	1.60	2.13	–
**4.45**	**a_2, a_3;OCH_2 (BD)**	**F-BD-DG**	–	0.31	0.85	1.16	3.89	13.80	–
**4.36**	**c; CH_2OCH_2 (DGA)**	**DG-BD-DG**	–	0.32	0.76	1.51	6.75	43.67	1.00
**4.30**	**a_1; OCH_2 (BD)**	**DG-BD-DG**	–	0.06	0.18	0.56	4.97	38.27	1.00
**1.90**	**b_4; OCH_2CH_2 (BD)**	**F-BD-F**	2.01	1.86	1.72	1.52	1.60	1.90	–
**1.83**	**b_2, b_3; OCH_2CH_2 (BD)**	**F-BD-DG**	–	0.33	0.85	1.29	3.93	11.89	–
**1.80**	**b_1; OCH_2CH_2 (BD)**	**DG-BD-DG**	–	0.05	0.13	0.59	4.94	38.76	1.01

Table S2. Comparison between the initial molar feed percentage and the real molar percentage of furanoate and diglycolate moieties.

(co)polymer	F/DG_{feed} (mol%)	F/DG (mol%)
**PBF**	100/0	100.0/0
**PBF-co-PBDG-**		
**90/10**	90/10	86.2/13.8
**75/25**	75/25	72.5/27.5
**50/50**	50/50	57.0/43.0
**25/75**	25/75	22.9/77.1
**10/90**	10/90	4.4/95.6
**PBDG**	0/100	0/100.0

1.2 ATR-FTIR

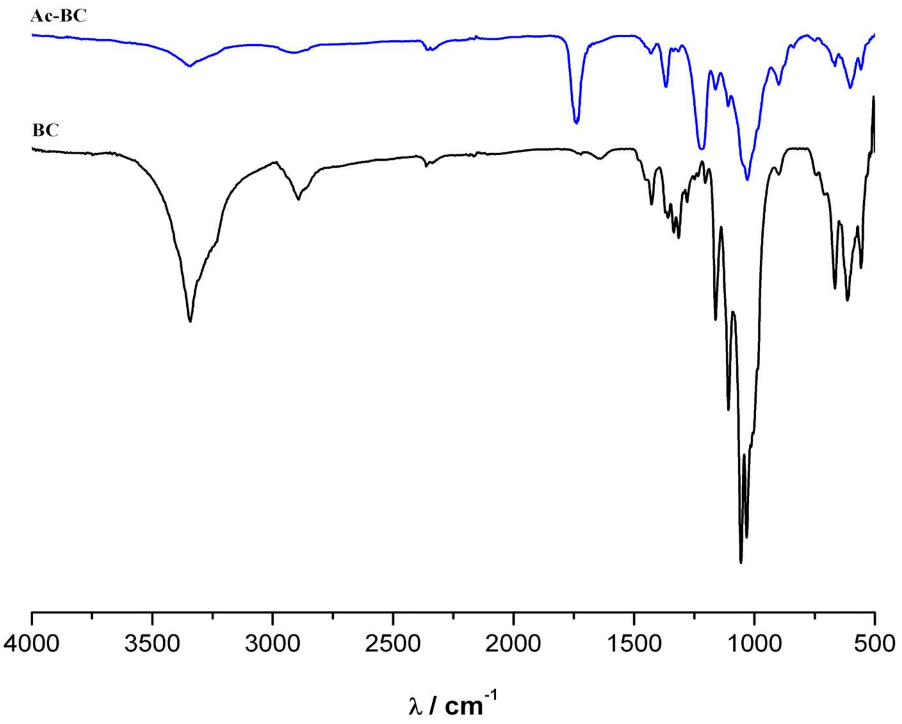

Figure S2. ATR FTIR spectra of the acetylated bacterial cellulose (Ac-BC) and of the unmodified bacterial cellulose (BC) fibres.

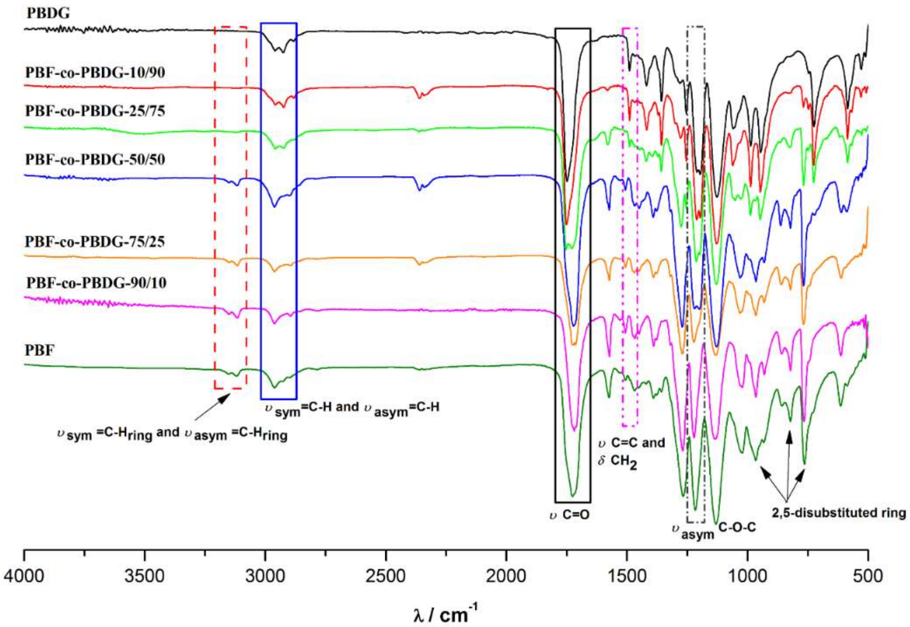

Figure S3. ATR FTIR spectra of PBF-co-PBDG copolyesters and of PBF and PBDG related homopolyesters.

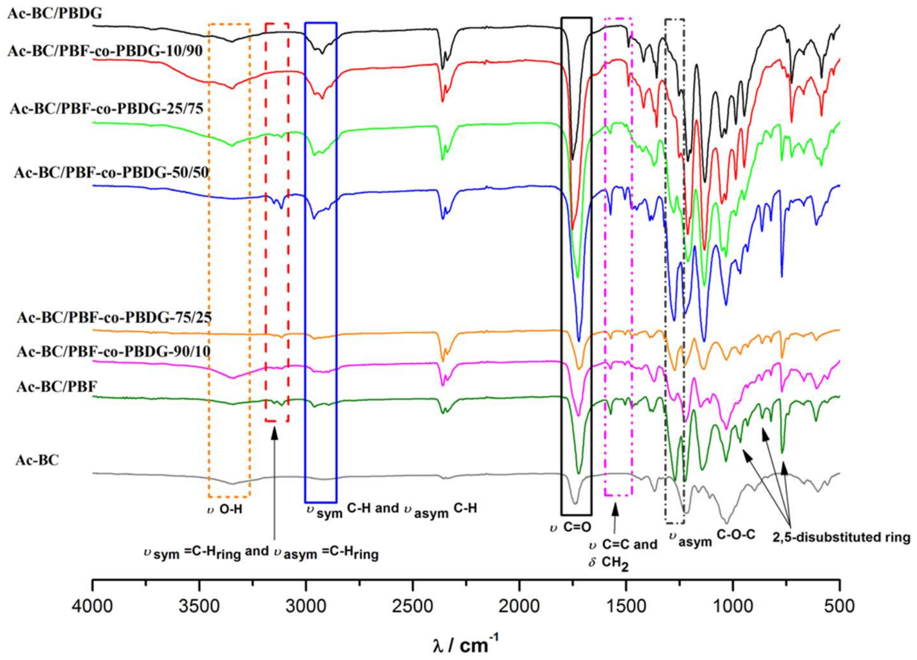

Figure S4. ATR FTIR spectra of all Ac-BC/PBF-co-PBDG nanocomposites.

1.3 SEM

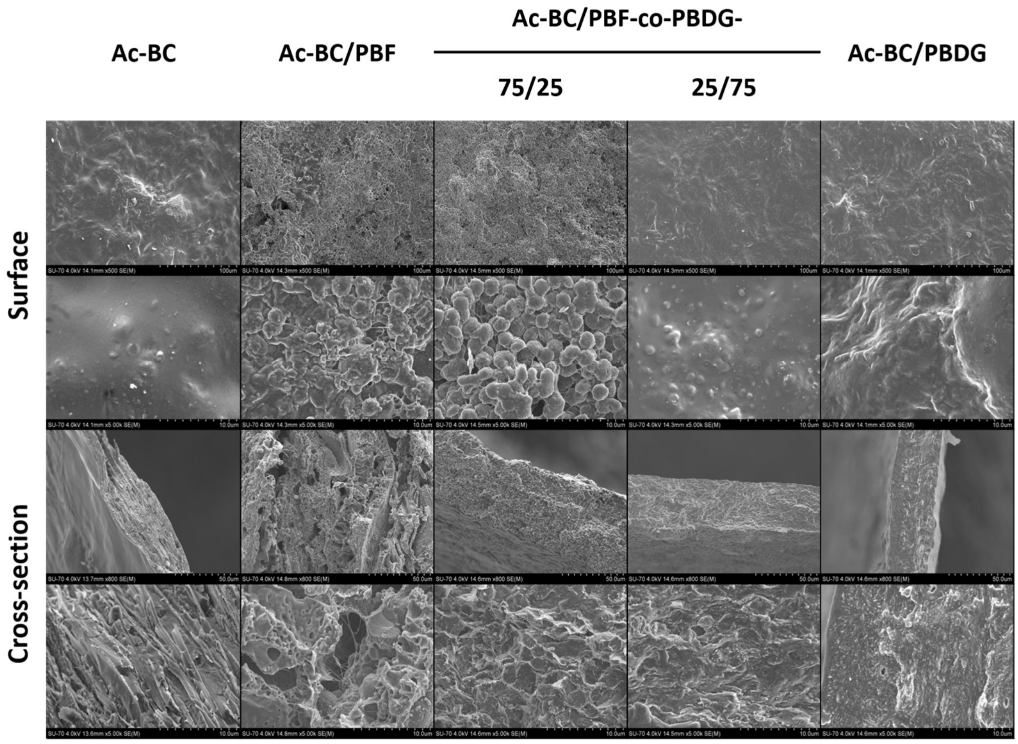

Figure S5. SEM micrographs of Ac-BC film and of the nanocomposites of the (a) surface (500 x and 5.0 kx) and (b) cross-section (800 x and 5.0 kx).

2. Contact angles with water

Table S3. Water contact angles of the composites films measured at several points in time for 40 s.

**Sample**	**CA_{water} / °**							
	**time / s**							
	**0**	**5**	**10**	**15**	**20**	**25**	**30**	**40**
**Ac-BC**	82.10 ± 1.93	71.09 ± 2.34	69.22 ± 3.05	67.93 ± 3.33	67.45 ± 3.03	66.83 ± 3.32	66.48 ± 3.42	65.97 ± 3.60
**Ac-BC/PBF**	116.40 ± 2.11	102.43 ± 5.03	101.96 ± 4.52	100.57 ± 5.37	98.14 ± 4.36	97.37 ± 4.05	96.89 ± 3.84	96.45 ± 4.04
**Ac-BC/PBF-co-PBDG-90/10**	105.10 ± 0.77	87.36 ± 2.41	83.01 ± 3.30	82.84 ± 3.30	82.41 ± 3.25	82.44 ± 3.59	82.04 ± 3.62	81.67 ± 3.57
**Ac-BC/PBF-co-PBDG-75/25**	101.85 ± 2.08	85.47 ± 3.87	78.85 ± 3.51	77.40 ± 3.70	75.97 ± 3.82	73.96 ± 3.62	73.61 ± 3.26	72.28 ± 3.82
**Ac-BC/PBF-co-PBDG-50/50**	86.97 ± 2.49	72.32 ± 2.75	69.39 ± 3.11	67.24 ± 2.81	65.30 ± 2.51	64.04 ± 2.67	63.62 ± 2.74	62.15 ± 2.75
**Ac-BC/PBF-co-PBDG-25/75**	74.29 ± 1.37	53.56 ± 3.82	50.50 ± 3.45	48.82 ± 3.15	47.46 ± 2.34	46.85 ± 2.21	46.26 ± 2.05	45.10 ± 1.66
**Ac-BC/PBF-co-PBDG-10/90**	70.40 ± 3.96	46.56 ± 1.71	45.48 ± 1.78	44.81 ± 1.78	44.61 ± 1.48	44.37 ± 1.70	44.27 ± 1.73	43.36 ± 1.83
**Ac-BC/ PBDG**	73.65 ± 1.67	54.04 ± 4.32	49.84 ± 2.73	48.18 ± 2.06	47.57 ± 1.37	46.74 ± 1.79	46.69 ± 1.95	45.96 ± 1.92

3. Crystallinity and thermal behaviour

3.1 X-ray diffraction (XRD) analysis

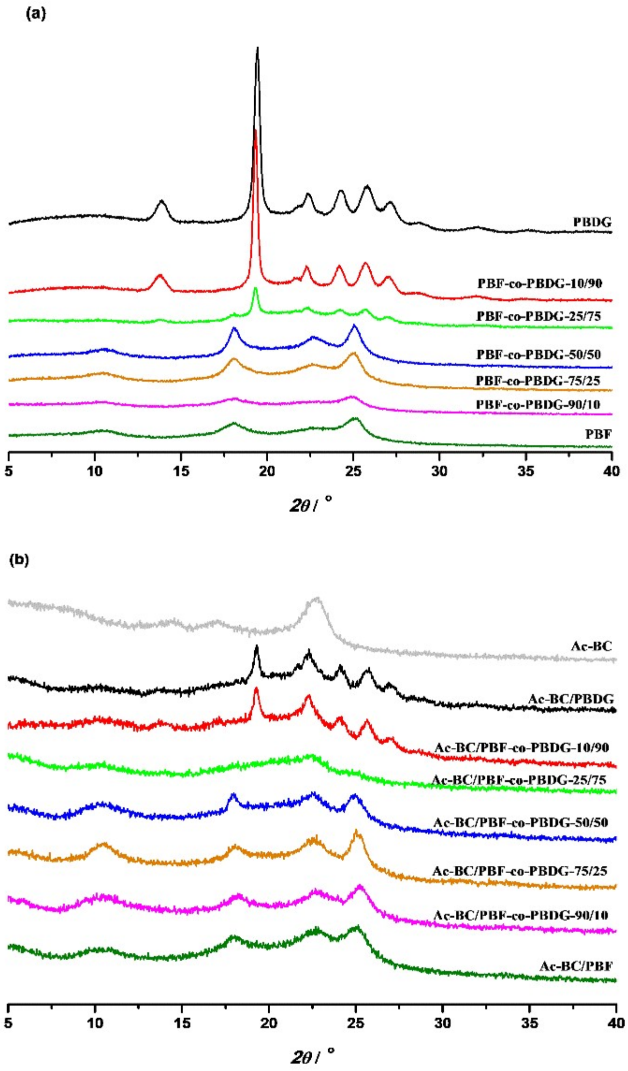

Figure S6. X-Ray diffractograms of the (a) neat (co)polyesters and (b) corresponding nanocomposites.

3.2 Differential scanning calorimetry (DSC)

Table S4. Important thermal values of the (co)polyesters and Ac-BC obtained by DSC and TGA analyses.

sample		T_g / °C	T_{cc} / °C	T_m / °C	$T_{d,\,5\%}$ / ºC	T_d / °C
**PBF**		46.1	-	173.9	348.7	380.5
**PBF-co-PBDG-**			-			
	**90/10**	25.1	-	161.7	328.6	368.4
	**75/25**	13.8	81.5	136.2	303.1	360.3
	**50/50**	-2.7	-	93.2	322.1	365.3
	**25/75**	-17.6	-	48.0	305.4	378.1
	**10/90**	-26.4	-	48.0	297.5	362.1
**PBDG**		-26.6	-	65.6	294.9	360.1
**Ac-BC**		-	-	-	278.2	363.0

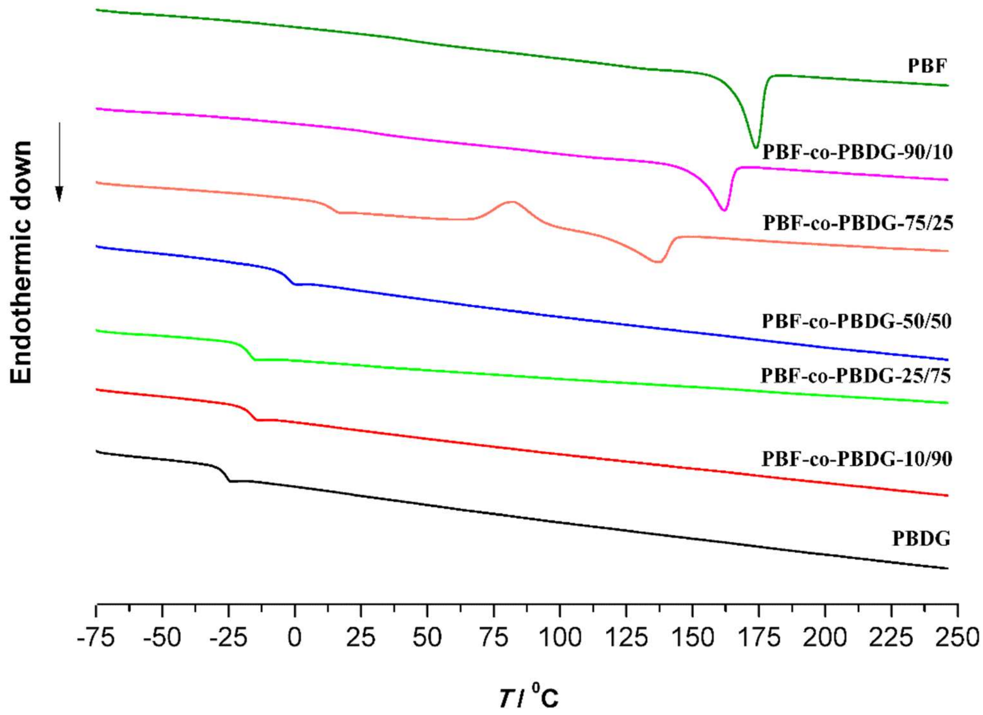

Figure S7. DSC traces of the PBF-co-PBDGs and related PBF and PBDG homopolyesters.

3.3 Thermogravimetric analysis (TGA)

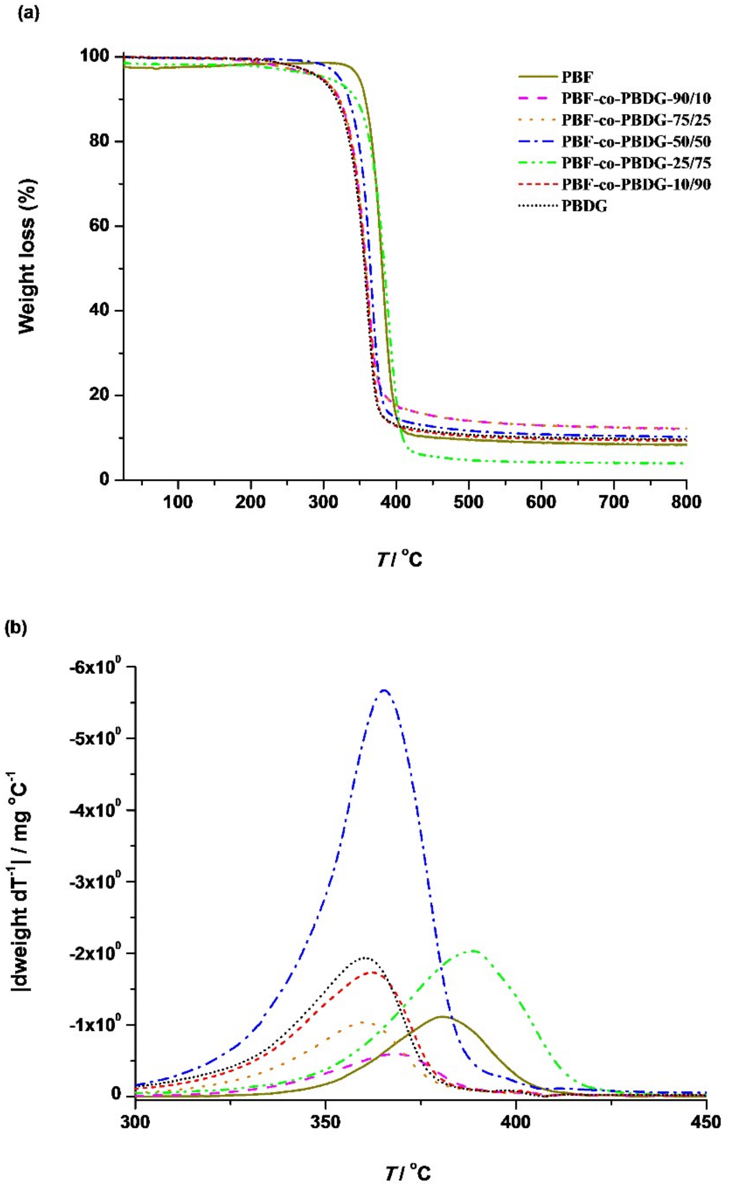

Figure S8. Thermogravimetric curves of the PBF-co-PBDGs and related PBF and PBDG homopolyesters: TGA (a) and (b) DTG.

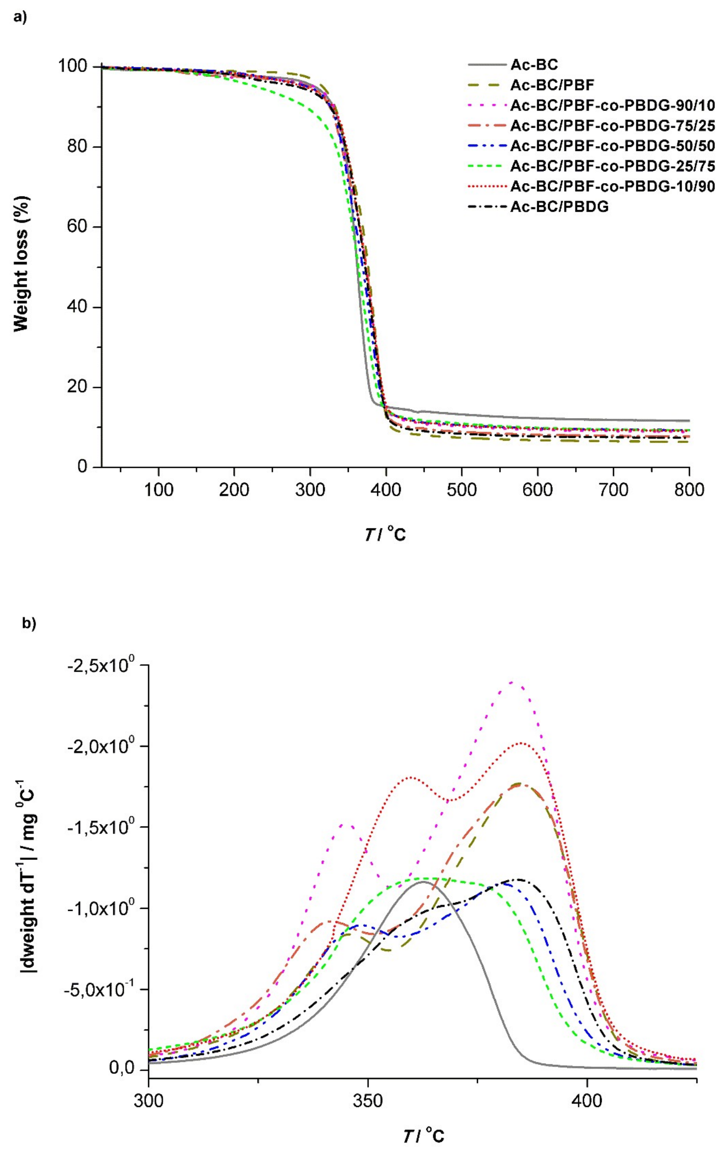

Figure S9. Thermogravimetric curves of the nanocomposites and Ac-BC: TGA (a) and (b) DTG.

3. Tensile tests

Table S5. Young's modulus, elongation at breakage and tensile strength of the nanocomposites and of Ac-BC component.

**sample** [1]	**Young's modulus / MPa**	**Elongation at break (%)**	**Tensile strength / MPa**
**Ac-BC**	1172.8	1.57	14.51
**Ac-BC/PBDG**	499.8	8.85	11.05
**Ac-BC/PBF-co-PBDG-**			
**90/10**	1239.3	0.62	7.62
**75/25**	447.8	0.99	6.32
**50/50**	360.2	7.19	7.36
**25/75**	30.3	25.02	6.22
**10/90**	374.4	7.28	8.07

[1] Ac-BC/PBF nanocomposite was not evaluated by tensile testing due to its brittle character, which broken easily precluding its test.
